# A Case of Extragenital Lichen Sclerosus: Beyond the Anogenital Region

**DOI:** 10.7759/cureus.101442

**Published:** 2026-01-13

**Authors:** Cynthia Estefania Fortozo Flores, Lucia Achell Nava, Guadalupe Maldonado-Colin, Mariela R Rosas Garcia, Marvin J Benavides Maruri

**Affiliations:** 1 Department of Dermatology, Universidad Nacional Autónoma de México, Mexico City, MEX; 2 Department of Dermatology, Centro Médico Nacional 20 de Noviembre, Instituto de Seguridad y Servicios Sociales de los Trabajadores, Mexico City, MEX; 3 Department of Pathology, Centro Médico Nacional 20 de Noviembre, Instituto de Seguridad y Servicios Sociales de los Trabajadores, Mexico City, MEX

**Keywords:** atrophic plaques, blaschko's lines, extragenital skin, inflammatory skin disease, lichen sclerosus

## Abstract

Lichen sclerosus et atrophicus is a chronic inflammatory dermatosis that predominantly affects the genital region. Its exclusively extragenital presentation is rare. We describe the case of a 78-year-old male patient with a history of colon cancer treated with chemotherapy and radiotherapy, who presented with lesions clinically, dermoscopically, and histopathologically consistent with extragenital lichen sclerosus. The patient received treatment with high-potency topical corticosteroids (0.05% clobetasol propionate) and calcineurin inhibitors (0.1% tacrolimus), achieving significant clinical improvement.

This case highlights the need to identify isolated clinical presentations of extragenital lichen sclerosus due to its low frequency, as well as to consider new therapeutic options for this disease.

## Introduction

Lichen sclerosus et atrophicus is a chronic inflammatory mucocutaneous disease of multifactorial etiology. Approximately 85% of cases involve the genital region, while only 15%-20% affect extragenital areas. Both presentations can coexist in most cases; however, isolated extragenital lichen sclerosus is a low-prevalence condition, occurring in only about 6% of cases [1]. Clinically, it is characterized by ivory-white papules that coalesce into sclerotic plaques with a parchment-like appearance. It commonly affects the trunk, abdomen, and extremities, and may be accompanied by purpura and telangiectasia [2]. Less frequent variants of extragenital lichen sclerosus have also been described, including Blaschko-linear lichen sclerosus, bullous lichen sclerosus, and hemorrhagic lichen sclerosus [3]. Diagnosis is based on clinical and dermoscopic findings, supported by histopathology. Differential diagnoses include vitiligo, hypopigmented mycosis fungoides, lichen planus, and morphea [2]. Treatment can be topical, intralesional, or systemic, depending on the extent of involvement and response to conventional therapy. High-potency topical corticosteroids are the cornerstone of treatment, while systemic therapies are reserved for extensive cases refractory to first-line management [2,3].

The objective of this report is to describe a clinical case of extragenital lichen sclerosus without genital involvement, to emphasize the need to identify isolated clinical presentations due to their low frequency, and to highlight its clinical characteristics, histopathological findings, and conventional and novel treatments, with the aim of expanding the existing medical literature.

## Case presentation

**Figure 1:**
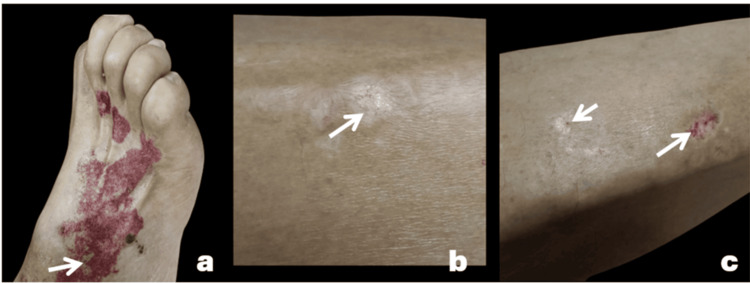
Clinical images of extragenital lichen sclerosus Atrophic pearly-white plaques are observed (a). Atrophic plaques with a "cigarette-paper" appearance (b) associated with peripheral purpuric macules (c).

A 78-year-old male patient with a history of colon cancer treated with chemotherapy and radiotherapy presented for evaluation of chronic white lesions on his legs. These were accompanied by purpuric lesions following trauma to the dorsum of the right foot and were associated with pruritus. Physical examination revealed a bilateral, asymmetrical dermatosis localized to the dorsum of the right foot and the lateral aspects of the legs, consisting of four atrophic plaques with a "cigarette paper" appearance of various sizes, the largest measuring 1 cm along its longest axis. The plaques were pearly white, oval-shaped, with well-defined erythematous-violaceous borders and were associated with peripheral purpuric macules (Figures 1a-1c).

**Figure 2:**
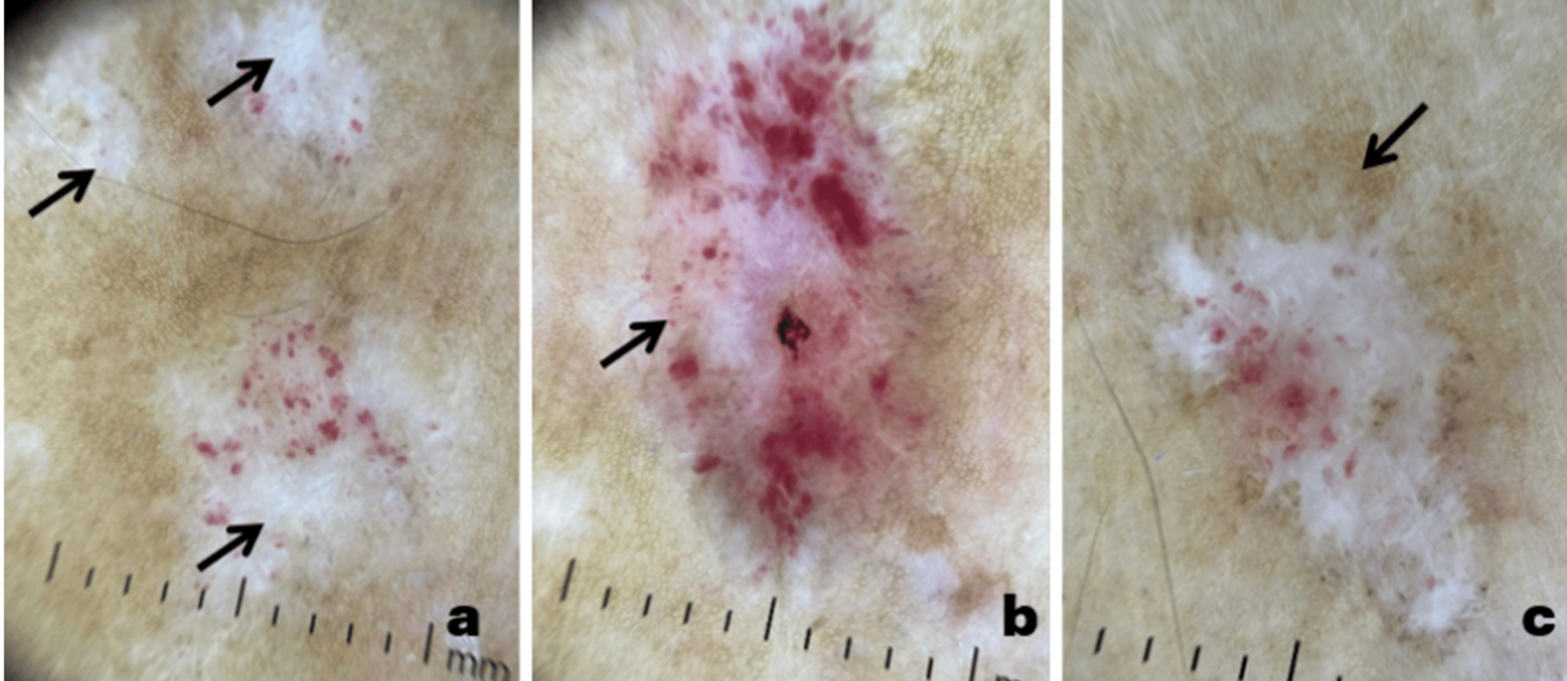
Dermoscopic images of extragenital lichen sclerosus Structureless white areas (a), purpuric spots (b), and peripheral pigment network (c) are observed.

Dermoscopy highlighted structureless white areas, purpuric spots, peripheral pigment network, comma- or dotted vessels, and whitish, fine lamellar scales (Figures 2a-2c).

**Figure 3:**
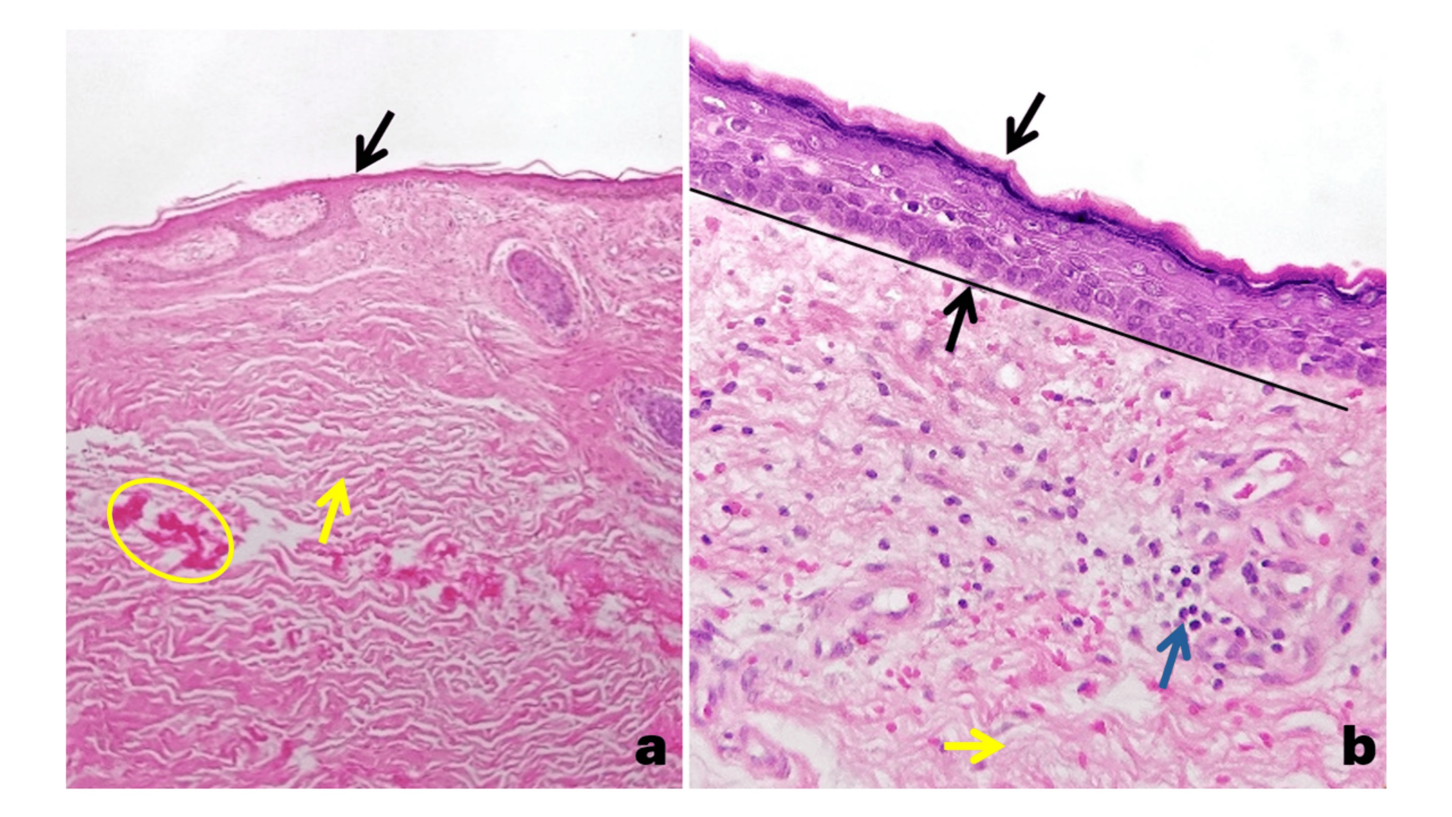
Histological images of extragenital lichen sclerosus stained with hematoxylin (a) Panoramic view (4×) of skin showing epidermal atrophy (black arrow), dermal fibrosis, collagen deposits (yellow arrow and oval), and a decrease in cutaneous adnexa. (b) At higher magnification (40×), compact orthokeratosis, epidermal atrophy (black arrow) with loss of the rete ridges (black arrow and line), fibrosis of the papillary dermis (yellow arrow), and a scant lymphocytic inflammatory infiltrate (blue arrow) are observed.

The patient had no anogenital or mucosal involvement. A skin biopsy reported epidermal atrophy, loss of rete ridges, dermal fibrosis with collagen deposition, decreased cutaneous adnexa, and sparse inflammatory infiltrate (Figures 3a-3b).

Based on the clinical and histopathological findings, a diagnosis of extragenital lichen sclerosus was made. Treatment was initiated with topical steroids and calcineurin inhibitors, resulting in clinical improvement of the lesions. No association with autoimmune diseases was found.

## Discussion

Lichen sclerosus is a chronic inflammatory mucocutaneous disease of multifactorial etiology [1]. Literature historically refers to lichen sclerosus cases as "atrophic," but in 1976, the term was replaced with simply "lichen sclerosus," as atrophy is not always observed histologically or clinically [1].

Extragenital lichen sclerosus has been associated with genetic factors such as HLA Class II DQ7, DQ8, and DQ9; autoimmune diseases, like vitiligo, alopecia areata, and Graves' disease, in approximately 34% of cases; infections by Borrelia, Epstein-Barr virus, and human papillomavirus (HPV); hormonal factors; and local factors, such as friction, trauma, surgery (due to the Koebner phenomenon in genetically predisposed individuals), and even following radiation therapy and sunburns [1,2]. Cases have also been reported in association with immune checkpoint inhibitors, such as ipilimumab and nivolumab [1].

The estimated prevalence ranges from 0.1% to 0.3%, with a bimodal peak (prepubertal patients and postmenopausal women, or men in their sixth decade of life) and a female predominance [4].

Clinically, the extragenital variant typically affects the upper trunk and extremities and has been reported in uncommon locations, such as the face (with nine case reports) and in patterns following Blaschko's lines [3,4]. The lesions begin as asymptomatic or mildly pruritic white papules that coalesce into ivory-white, atrophic plaques [2].

The diagnosis is clinical and confirmed by histopathology [2]. Current treatment options are limited. First-line management may include localized therapy (with high-potency topical or intralesional corticosteroids, retinoids, vitamin D analogs, and calcineurin inhibitors) or systemic therapy (retinoids, corticosteroids, methotrexate, cyclosporine, hydroxyurea, phototherapy with psoralen plus ultraviolet A, or tacrolimus and narrowband ultraviolet B), depending on the extent of disease and treatment resistance [1-4].

A novel therapeutic alternative with the use of Janus kinase (JAK) inhibitors has recently been documented [5]. A systematic review conducted by Shen et al. included 43 patients with lichen sclerosus, of whom four had the extragenital variety. They were treated with systemic or topical JAK inhibitors (abrocitinib, baricitinib, and ruxolitinib), showing improvement in symptoms, clinical lesions, and quality of life in both genital and extragenital lichen sclerosus [5]. The rationale for this therapy is based on JAK inhibitors interfering with the interferon (IFN)-gamma-JAK/signal transducer and activator of transcription (STAT) signaling pathway, which is a pathogenic pathway shared by lichen sclerosus and other autoimmune diseases, such as vitiligo or morphea. Although evidence for the use of JAK inhibitors in extragenital lichen sclerosus is limited to case reports, it opens the door for future randomized controlled trials [5].

Long-term follow-up of this entity is necessary, as extragenital lichen sclerosus can coexist with the genital variety, which carries a 5% risk of malignancy [1]. In general, extragenital lichen sclerosus lacks carcinogenic potential; however, Sergeant et al. reported two cases with transformation to squamous cell carcinoma, a process perhaps promoted by chronic inflammatory status and the prolonged use of immunosuppressive medications [6].

This case is relevant, as it involves a patient with a history of radiation and trauma, which are known triggering factors, thus supporting the theories involved in its pathogenesis. Given that extragenital lichen sclerosus associated with the genital variety is a low-frequency entity, and isolated presentations are even rarer, it is crucial to document such cases to expand the clinical spectrum and the current therapeutic options available for this disease.

## Conclusions

Extragenital lichen sclerosus can present in isolation in approximately 6% of cases and has a multifactorial etiology; trauma and radiotherapy are triggering factors that should be considered. Several lines of treatment exist, with topical steroids being the first line. However, for extensive and resistant cases, systemic treatments, such as JAK inhibitors, have demonstrated clinical and symptomatic remission. These are used off-label, and current evidence is primarily limited to case reports and series; therefore, randomized controlled trials are needed.
